# Exploring perspectives of Dscam for cognitive deficits: a review of multifunction for regulating neural wiring in homeostasis

**DOI:** 10.3389/fnmol.2025.1575348

**Published:** 2025-05-02

**Authors:** Yinyi Xiong, Li Li, Xiaorong Zhang

**Affiliations:** ^1^Department of Rehabilitation, Affiliated Hospital of Jiujiang University, Jiujiang, China; ^2^Department of Intensive Care Unit, Affiliated Hospital of Jiujiang University, Jiujiang, China; ^3^Department of Pathology, Affiliated Hospital of Jiujiang University, Jiujiang, China

**Keywords:** Dscam, homeostatic synaptic plasticity, cognitive deficits, learning and memory, Alzheimer’s disease

## Abstract

Down syndrome cell adhesion molecule (Dscam) represents a group of cell surface transmembrane receptors with a conserved protein structure across species. In *Drosophila*, Dscam exhibits extensive isoform diversity resulting from alternative splicing, providing each cell with a unique identity. Identical isoforms expressing on the surfaces of opposing cells mediate homophilic interactions, thereby driving intracellular signaling for establishment of complex neuronal branching patterns. Mammalian Dscam lacks isoform diversity but retains the homophilic binding property. In contrast, it is capable of mediating multifaced neurological functions which are more complex than those of *Drosophila* Dscam. In this review, we spotlight that the homeostatic mechanisms mediated by Dscam are significant for normal cognitive function. Down syndrome (DS) and autism spectrum disorders (ASD) are two common neurodevelopmental diseases, the cognitive deficits of which are frequently correlated with aberrant DSCAM expression. Previous studies have presented some evidence that the neural homeostatic mechanisms associated with DSCAM are compromised in these two diseases. However, the insight into DSCAM-mediated homeostatic plasticity remains seriously overlooked. Furthermore, recent studies put forward that DSCAM might be one of the key molecules involved in neuronal age-related mechanisms during early stage of Alzheimer’s disease (AD), a neurodegenerative disease linked to aberrant homeostatic mechanisms. In this review, we aim to provide a comprehensive understanding of Dscam-mediated crucial roles in regulating neural circuitry for homeostasis, thus elucidating how Dscam induces changes of homeostatic plasticity to affect cognitive function in either physiological or pathological conditions. We hope this review could inspire future studies to test the extent to which Dscam-mediated neural homeostatic mechanisms contribute to neurological disorders accompanied by cognitive deficits, thus facilitating research on discovering potential therapeutic avenues.

## Graphical abstract

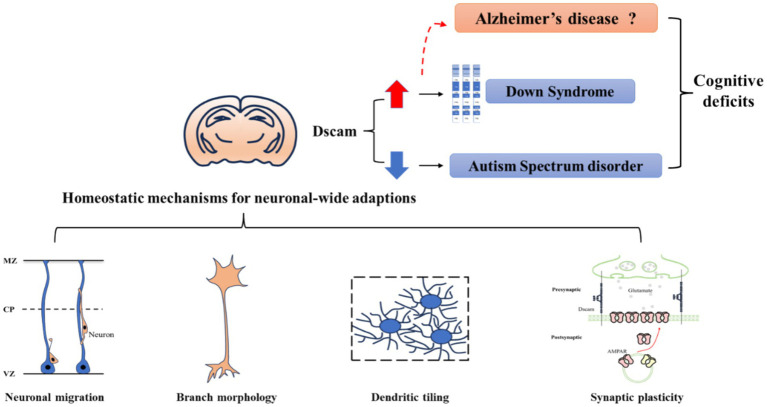

## 1 Introduction

The *Down syndrome cell adhesion molecule* (*Dscam*) is a gene that encodes the largest member of immunoglobulin superfamily of cell adhesion molecules in both vertebrates and invertebrates (Graveley et al., 2004; Crayton et al., 2006). In *Drosophila*, there are four genes encoding Dscam: *Dscam1*, *2*, *3*, and *4*. Among these genes, *Dscam1* has been most extensively examined. A range of studies have demonstrated that *Dscam1* is subject to an alternative splicing mechanism, whereas *Dscam2*, *Dscam3*, and *Dscam4* do not undergo such a large-scale alternative splicing (Neves et al., 2004; Schmucker et al., 2000; Graveley, 2005; Millard et al., 2007; Hattori et al., 2008). The alternative splicing of *Dscam1* is temporally and spatially regulated, resulting in stochastic but biased expression of *Dscam* splice variants in individual cells (Neves et al., 2004; Celotto and Graveley, 2001). In vertebrates, the *Dscam* gene, isolated from syntenic region for human chromosome band 21q22.2-22.3, is simpler compared to its homolog of *Drosophila Dscam*, as it does not undergo alternative splicing of exons (Crayton et al., 2006; Agarwala et al., 2001a).

The Dscam protein is a group of cell surface transmembrane receptors, and provides individual cells with a unique identity by which a cell can distinguish itself from others (Hattori et al., 2007; Wojtowicz et al., 2007). The structure of Dscam is highly conserved across species, consisting of 10 immunoglobulin (Ig) domains, 6 fibronectin type III (FNIII) domains, a transmembrane (TM) region and a cytoplasmic domain (Stachowicz, 2018; Hizawa et al., 2024; Barlow et al., 2001). In *Drosophila*, as many as 38,016 Dscam isoforms are generated, and each isoform consists of an ectodomain containing a unique combination of three different variable Ig domains linked to one of two alternative transmembrane segments (Schmucker et al., 2000). Further studies demonstrated that each isoform binds strongly to itself, but weakly to other isoforms (Wojtowicz et al., 2007; Wojtowicz et al., 2004; Hughes et al., 2007; Meijers et al., 2007; Sawaya et al., 2008). Therefore, Dscam isoform diversity provides a molecular mechanism for selective recognition among neurons, contributing to the complexity and specificity of neuronal wiring (Wojtowicz et al., 2007; Wojtowicz et al., 2004; Miura et al., 2013). In contrast, mammalian Dscam lacks diverse isoforms, but retains the homophilic binding property for axon segregation and dendrite repulsion. This characteristic of mammalian Dscam underscores the necessity of alternative splicing on other cell adhesion molecules to compensate the lack of cell–cell differences defined by Dscam diversity (Huang et al., 2011; Garrett et al., 2018). Mammalian Dscam can either bind to diffusible chemorepellent or chemoattractant for neurological function. Moreover, mammalian Dscam can be released from cell surface membrane through proteolytic cleavage. As a result, the intracellular domain of Dscam can translocate into nucleus and trigger signaling related to neural process (Sachse et al., 2019).

Multiple lines of evidence suggest that Dscam is expressed in specific areas of the developing central nervous system (Yamakawa et al., 1998). The expression profile of Dscam in mice has revealed that Dscam is highly expressed in the cerebral cortex, hippocampus, olfactory bulb, thalamus and cerebellum, areas crucial for advanced cognitive functions like learning, memory, sensory perception and voluntary movement (Agarwala et al., 2001a; Barlow et al., 2001). Reports show that aberrant Dscam expression is implicated in the cognitive deficits of several neurodevelopmental disorders. In Down syndrome (DS), Dscam overexpression is associated with intellectual disability and verbal memory deficit in patients and impaired cognitive performances in mouse models (Stachowicz, 2018; Curtis et al., 2023). Similarly, *DSCAM* loss-of-function mutations show a relatively high frequency in autism spectrum disorder (ASD) patients, and Dscam deficiency leads to abnormal voluntary locomotor control and ASD-like behaviors in mouse models (Lemieux et al., 2016; Laflamme et al., 2019; Xu et al., 2011; Chen et al., 2022; Lim et al., 2021). A recent study on adult worker honey bees shows that reducing Dscam expression enhances memory after olfactory reward conditioning (Ustaoglu et al., 2023). Collectively, these cross-species evidences underscore Dscam’s importance in cognitive processes, making it a prime candidate for research on cognitive deficits.

Homeostatic plasticity is widely defined as a mechanism by which neurons tend to move back toward an original state (Kavalali and Monteggia, 2023). It works opposite to Hebbian plasticity, a mechanism for neurons to encode and retain information. There has been a consensus that integrating both Hebbian and homeostatic plasticity is conductive to achieve a comprehensive understanding of memory formation and retention (Fox and Stryker, 2017). Since appropriate expression levels of Dscam are fundamental for normal neural development, it is considered to participate in neural homeostatic mechanisms. Through synaptic, neuronal and network-wide adaptations, Dscam functions as a key regulator to stabilize neuronal activity triggered by activity experiences. Therefore, the insight into Dscam-mediated homeostatic mechanisms could provide an in-depth understanding of the neurological basis of cognitive function.

In this review, we focus on Dscam-mediated homeostatic mechanisms by reviewing the neurological functions of Dscam obtained from studies across species. Subsequently, we lay out evidence associating aberrant Dscam expression with neurological disorders like DS and ASD. Finally, noticing a potential role of Dscam in neurodegeneration suggested by recent findings, we put forward a speculative hypothesis that Dscam may be one of the pivotal molecules engaged in the aging processes that occur during the early stage of Alzheimer’s disease (AD). Taken together, we aim to offer valuable insights into cognitive deficits for future study and underscore the therapeutic capability of targeting Dscam in disorders featuring cognitive deficits.

## 2 The neurological function of Dscam: how does Dscam contribute to the homeostasis of the neural network underpinning cognitive function

A great many studies have shown that Dscam is fundamental for neural development through its multiple functions. The Dscam-mediated homeostatic mechanisms can operate at a synaptic, neuronal, and network scale, thereby contributing to the cognitive function. Here, we discuss the Dscam-mediated homeostatic mechanisms from the following three aspects: neuronal migration, neuronal morphology and patterning, and synaptic specificity and plasticity.

### 2.1 Neuronal migration

In the cortex of adult mice, Dscam is predominately expressed in the layer III/V pyramidal cells (Barlow et al., 2002). Analysis of early postnatal mice harboring a spontaneous *Dscam* mutation revealed a diminished thickness in the upper cortical layers II/III. This subtle change in cortical lamination is transient, and could not be attributed to changes in cell number (Maynard and Stein, 2012). Therefore, it leads to an initial speculation that Dscam is involved in neuronal migration. To further investigate the migration defect of cortical neurons caused by Dscam deficiency, researchers transfected the neuronal progenitors with short hairpin RNA (shRNA) vectors targeting *Dscam* in mouse embryos, and then analyzed these mice after birth. The results revealed a migration delay effect in Dscam deficient cortical neurons (Zhang et al., 2015). Since Dscam deficiency does not change the cell fate of migratory neurons, the thickness of cortical layers II/III was reduced in early postnatal stages but was later restored by adulthood (Maynard and Stein, 2012; Zhang et al., 2015). Similarly, knockdown of *Dscam* impaired endfeet detachment of newborn neurons and their subsequent radial migration in the mouse dorsal midbrain (Arimura et al., 2020). Collectively, these experimental results demonstrate an essential role of Dscam in neuronal migration. Remarkably, this Dscam-mediated effect on neuronal migration might be restricted to a specific period or a subpopulation of neurons. Unlike postnatal cortical development and embryonic midbrain delamination in *Dscam* mutant mice, the embryonic cortical lamination as well as interneuron migration are unaffected by Dscam loss-of-function (Mitsogiannis et al., 2020). Indeed, the Dscam-regulated neuronal migration is an important component of its homeostatic roles in the nervous system, as evidenced by migration deficits caused by Dscam gain-of-function. For example, the radial migration of both mouse pyramidal neurons and cortical interneurons is compromised by overexpression of Dscam (Mitsogiannis et al., 2020). Consistently, the migration capacity in 2D culture systems is decreased in GABAergic interneurons differentiated from DS induced pluripotent stem cells (iPSCs) (Huo et al., 2018).

Previous studies provide some explanations related to the mechanisms responsible for Dscam-mediated neuronal migration. Before the initiation of migration, nascent neurons should detach their apical endfeet from the proliferative zone (Hatakeyama et al., 2014). There is no direct evidence to support that the trans-Dscam homophilic interaction between newborn neurons and radial glia is responsible for endfeet detachment of newborn neurons (Arimura et al., 2020). Instead, transcriptional repression of the cadherin family in delaminating cells is of importance for neuronal delamination, which is usually discussed in the epithelial-mesenchymal transformation model (Arimura et al., 2020; Hatakeyama et al., 2014). Rap1 activity is crucial for apico-basal polarity of epithelial cells by regulating the membrane localization of N-cadherin. In the mouse dorsal midbrain, Dscam inhibits the RapGEF2-Rap1 pathway in nascent neurons attached to the ventricular surface, and subsequently regulates the N-cadherin localization which is required for the departure of cells from the ventricular surface (Arimura et al., 2020). The Dscam-N-cadherin interaction is also required for migrating neurons at the end of migration. A study has identified that Dscam deficiency may enhance the N-cadherin-mediated cell adhesion within the upper cortical plate, leading to thinner upper cortical layers (Yang et al., 2022). Furthermore, as suggested by transcriptional analysis of human induced GABAergic neurons, identification of key regulators within the downstream cell motility-related pathways could also facilitate the mechanistic understanding of Dscam-mediated neuronal migration (Huo et al., 2018). Researchers found that the activity of Pak1, a critical kinase related to the actin cytoskeleton pathway, is upregulated by overexpression of Dscam (Pérez-Núñez et al., 2016). Given that the migration defects of GABAergic neurons induced from DS patients could be partly rescued by the application of a Pak1 inhibitor, it is believable that Pak1 is a key regulator for neuronal migration (Huo et al., 2018).

Although these mechanistic studies remain superficial, the Dscam-mediated neuronal migration ensures that neurons are destined for proper layers and form precise synaptic connections. Therefore, it provides foundation for regulating neural homeostasis on a network-wide scale, thereby facilitating an internal topographic representation of the external world.

### 2.2 Neuronal morphology and branch patterning

The role of neuronal morphology and branch patterning mediated by Dscam is extensively examined by a great number of studies. Dscam is dynamically regulated with neuronal differentiation during development (Agarwala et al., 2001a; Bruce et al., 2017). Given the subcellular localization, Dscam is potentially involved in the axonal and dendritic processes, and subsequently contributes to the formation of neuronal morphology (Bruce et al., 2017; Soba et al., 2007; Agarwala et al., 2001b; Ly et al., 2008). Based on the neurological function of Dscam, the graded Dscam distribution in the nervous system makes it a strong candidate for topographic mapping (Santos et al., 2022).

Dscam is required for axon outgrowth. This view is strongly supported by genetic loss-of-function and gain-of-function studies across species (Schmucker et al., 2000; Bruce et al., 2017). It is further suggested that Dscam mediates axon outgrowth in a dose-sensitive manner. The direct evidence stems from knockdown experiments on *Drosophila* motoneurons, where the dendritic growth is initially augmented, and subsequently becomes impaired with a further increase in knockdown strength (Hutchinson et al., 2014). Strikingly, when the axonal growth is impaired by *Dscam* mutation, the path of axon projection away from initial regions is not affected in both *Drosophila* antennal olfactory receptor neurons (ORNs) and mouse retinal ganglion cells (Bruce et al., 2017; Hummel et al., 2003). In contrast, researchers observed that ORNs exhibit axonal targeting defects, with redundant branches terminated in ectopic locations (Hummel et al., 2003; Wang et al., 2002). These observations suggest that Dscam is not only essential for axon outgrowth, but also plays an important role in targeting specificity. With the role of Dscam for axon outgrowth and targeting specificity, neurites could extend along anticipated directions and establish precise synaptic connections (Simmons et al., 2017). Normal axon projection and correct lamina targeting would ensure the formation of different neural circuits (Millard et al., 2007).

In addition, Dscam is also required for dendritic arborization and spacing across species (Wang et al., 2002; Fuerst et al., 2008). This role of Dscam is supposed to be closely associated with its homophilic repulsion (Soba et al., 2007; Millard and Zipursky, 2008; Liu C. et al., 2020). Through homophilic repulsion and isoform diversity of Dscam, *Drosophila* neurons can evenly occupy a specific area without self-crossing but overlap with different types of neurons. Although mammalian Dscam lacks isoform diversity, it does mediate self-avoidance at the level of individual cell, homotypic cells, and heterotypic cells through homophilic interactions. This model is further suggested by recognitive and repulsive mechanisms different from those of *Drosophila*.

Overall, this function ensures processing of synaptic inputs for homeostasis of the neural system by regulating neuronal morphology and neural wiring. For a better understanding of the underlying mechanisms, we summarize previous findings into the following aspects:

#### 2.2.1 Homophilic repulsion

In *Drosophila*, loss of Dscam perturbs segregation of axonal branches in mushroom body (MB) neurons and causes dendritic self-crossing and branch fasciculation in dendrite arborization (da) neurons (Hughes et al., 2007; Soba et al., 2007; Matthews et al., 2007). The gain-of-function studies and rescue experiments further demonstrated that single Dscam isoforms are sufficient to mediate recognition between cell surfaces and subsequently initiate self-avoidance of dendrites (Soba et al., 2007; Matthews et al., 2007). Either the Dscam isoform diversity or any specific isoform is not required for dendritic self-avoidance in individual neurons, since no self-avoidance defects are caused by alleles with deleted exon 4 (Matthews et al., 2007). We called this mechanism for dendritic self-avoidance in *Drosophila* homophilic repulsion, where neurites distinguish sister branches from those of other cells through interactions between identical Dscam isoforms on the cell surface (Zinn, 2007). Likewise, retinal cells in *Dscam* mutant mice display hyperfasciculated processes, indicating that homophilic repulsion is a universal mechanism of Dscam across species (Fuerst et al., 2008; Fuerst et al., 2009).

Several lines of evidence suggest that the cytoplasmic domain of Dscam is essential for pathways that convert homophilic interactions to self-avoidance (Matthews et al., 2007). It is reported that the phosphorylation of Pak1 downstream of Dscam plays an important role in neuronal branching. However, it appears not necessary for Dscam-mediated repulsion, because the absence of endogenous Pak cannot result in observable dendritic self-avoidance defects in *Drosophila* da neurons (Hughes et al., 2007). Similarly, the intracellular Hpo/Trc/Fry pathway is also not involved in self-avoidance in da neurons (Soba et al., 2007). Identification of additional substrates for mediating Dscam’s repellent signaling is required.

It is revealed that the homophilic repulsion mechanism is also essential for tiling, a phenomenon that homotypic neurons respect the territory of each other for preserving the spatial information of synaptic inputs (Millard et al., 2007; Fuerst et al., 2012). In the absence of Dscam, the neuronal processes often fasciculate with each other, and then pull the cell bodies out of their mosaic position (Fuerst et al., 2008). This observation suggests that Dscam-mediated self-avoidance promotes complete territory coverage by counterbalancing extrinsic self-association signals. In *Drosophila*, these signals can be provided by netrin-B and frazzled (Matthews and Grueber, 2011). However, previous studies showed that Dscam1 deficiency may cause separate defects in dendritic self-avoidance and tiling in some specific types of neurons (Hughes et al., 2007; Soba et al., 2007; Hutchinson et al., 2014). Furthermore, phenotyping studies on retinal cells in several strains of *Dscam*-mutant mice observed either cell spacing defects in the absence of fasciculation or abnormal morphology with normal spacing (Fuerst et al., 2008; Fuerst et al., 2012). Therefore, self-avoidance and tiling, no matter in vertebrates or invertebrates, are mutually independent phenotypes (Fuerst et al., 2012).

It is thought that they may differ in their recognition and repulsion mechanisms based on Dscam. Multiple lines of evidence suggest that Dscam1 is not required for mediating tiling in all types of neurons in *Drosophila*, like motoneurons, Class IV da neurons (Soba et al., 2007; Hutchinson et al., 2014). By examining phenotypes of dendrite growth and spacing in eight different types of neurons, the study demonstrates that Dscam1 exerts its function in a cell-type-specific manner (Wilhelm et al., 2022). A study also reported that Dscam2-mediated homophilic repulsion is required for tiling in L1 and L2 lamina neurons in the *Drosophila* visual system (Millard et al., 2007; Lah et al., 2014). Strikingly, phenotypes of L1 or L2 neurons are specific to one of the two alternative isoforms of Dscam2 (Lah et al., 2014). Collectively, these findings suggest that the neuronal tiling requires specific isoforms of Dscam which is different in each cell type of neuron (Fuerst et al., 2012; Wilhelm et al., 2022; Lah et al., 2014).

For mammalian Dscam, its deficiency in a specific cell class can reproduce *Dscam*-mutant phenotypes to varying degrees (Fuerst et al., 2012). Since researchers observed that an intermediate Dscam dosage disrupts cell spacing but not arborization of dopaminergic retinal cells, mammalian Dscam potentially acts in a dose-dependent manner for differently regulation of self-avoidance and tiling (Fuerst et al., 2012). The interactions between Dscam and its environment are also essential for the function of mammalian Dscam (Fuerst et al., 2012). It likely responds to certain molecules within the environment to modulate branch positioning and cell spacing. For example, mammalian Dscam-mediated homophilic repulsion could serve as a general nonstick signal to counteract the adhesive responses mediated by multiple cell adhesion molecules (Garrett et al., 2018). Furthermore, the Dscam-mediated repulsive mechanisms underlying self-avoidance and tiling could either be different, as previously mentioned Trc kinase which is not required for self-avoidance, but mediates tiling in *Drosophila* (Zinn, 2007).

Although homophilic repulsion ensures that an individual neuron processes arborize to evenly fill a particular spatial domain, different types of neurons often have overlapping dendritic fields. This is essential for allowing different neuronal types to process different aspects of inputs. In *Drosophila*, overexpression of identical Dscam isoform in both of neighboring neurons could strongly decreased the area of overlapping dendritic fields, indicating that Dscam-mediated homophilic repulsion participates in the interactions between different neurons (Hughes et al., 2007; Soba et al., 2007; Matthews et al., 2007). The isoform diversity of Dscam is an important strategy for neighboring neurons in *Drosophila* to extensively overlap with each other. Each neuron expresses a unique combination of isoforms, allowing neurites to recognize self and nonself. Mammalian Dscam promotes self-avoidance at the level of individual cell without extreme isoform diversity. It has been shown that different types of amacrine neurons mutated for Dscam do not influence each other’s spacing. Therefore, additional isoform-rich molecules are considered to be involved in complex recognition procedures in vertebrates. Cell adhesion molecules like cadherins and protocadherins may serve as good candidates.

#### 2.2.2 Adhesive mechanisms

Studies have shown that Dscam is essential for axon outgrowth. For example, Dscam deficiency in mice impairs the axonal growth of retinal ganglion cell (RGC), leading to a delay in reaching the dorsal thalamus. In contrast, overexpression of Dscam causes exuberant growth in to the dorsal thalamus (Bruce et al., 2017). Further studies indicated that the Dscam’s role of axon growth may be likely explained by Dscam-mediated adhesive mechanisms rather than canonical self-avoidance. Mammalian Dscam does not undergo alternative splicing, but it can be constitutively cleaved from neuronal cell surface. As a result, the N-terminal portion sheds into the extracellular environment. By using a chimeric culture system, it is demonstrated that Dscam in both neuronal axons and their environment is essential for axon growth *in situ* (Bruce et al., 2017). This finding indicates that the homophilic interaction of Dscam, which underlies axon growth, is likely to mediate adhesion but not repulsion.

In *Drosophila*, *Dscam* mutants can impair axon pathways in the developing embryos, similar to the phenotypes in Netrin mutants. The dose-sensitive interactions between Dscam and netrin-1 suggest Dscam can act as a netrin receptor for axon guidance. Another study revealed that Dscam can direct axon-pathing through the interactions between Dscam, Dock, and Pak. In detail, Dscam can directly bind to the adaptor protein Dock, forming a Dscam-Dock complex. When Dscam undergoes tyrosine phosphorylation, the complex experiences a conformational change, thereby facilitating the recruitment of Pak1 to the cell membrane. As a result, Dscam can modulate cytoskeleton dynamics for axon guidance (Schmucker et al., 2000; Muda et al., 2002). Further study indicates that the Dscam-mediated membrane localization of Pak1 defines the stereotypical dendrite growth site in the *Drosophila* aCC motoneurons (Kamiyama et al., 2015). Multiple lines of evidence suggest that the netrin/Dscam signaling can be a generalized mechanism for axon guidance in both vertebrates and invertebrates (Ly et al., 2008; Andrews et al., 2008). However, there is no direct evidence to support the presence of Dscam-Dock-Pak signaling in vertebrates.

The netrin/Dscam signaling has also been extensively studied in vertebrates. Netrin-1 is a bifunctional guidance cue in the extracellular environment, and induces attractive or repulsive signals depending on their receptors (Yamagishi et al., 2020). Dscam expressed on axons can directly bind to netrin-1, thereby directing axons to grow and across the midline of mammalian CNS system (Ly et al., 2008; Andrews et al., 2008). Remarkably, this mechanism may not act in chick spinal interneurons (Cohen et al., 2017). Deleted in colorectal cancer (DCC) can also mediate attractive responses to netrin-1 in a way similar to Dscam. Further studies demonstrated that Dscam and DCC exhibit identical binding affinity to netrin-1, and functions independently in netrin-1 signaling (Ly et al., 2008; Andrews et al., 2008; Liu et al., 2009). Both of the two signaling pathways coordinate in parallel for normal axon guidance in mice (Palmesino et al., 2012). On the other hand, Dscam can interact with UNC5 to elicit repulsion in response to netrin-1 for growth cone collapse (Purohit et al., 2012). Furthermore, reports show that downstream of both the Dscam/DCC and Dscam/UNC5 pathways in netrin-1 signaling converges on regulating the microtubule dynamics. This occurs through the direct interactions of TUBB3 with Dscam, DCC, and UNC5 (Huang et al., 2015; Shao et al., 2017; Qu et al., 2013a).

Given the coupling of Dscam and microtubule dynamics, the functional significance of the interaction between the C-terminus of Dscam and multi-PDZ-domain-containing scaffolding proteins is selectively studied. The results showed that this Dscam/PDZ interaction is required to mediate a subset of Dscam’s function in specific cell types, indicative of differential dependence on PDZ-interacting domain of Dscam across cell types (Garrett et al., 2016). In previous studies, the cytoplasmic domain of Dscam and diverse protein kinases are demonstrated to be important for mediating netrin signaling across several neuronal culture systems (Liu et al., 2009; Purohit et al., 2012; Huang et al., 2015; Zhu et al., 2013; Qu et al., 2013b). Dscam is capable of activating Pak1, JNK, and p38MAP kinases. For the commissural neurons, Dscam can phosphorylate Fyn and Pak1 to promote netrin signaling (Liu et al., 2009). In another study, activation of JNK1 is important for netrin-1signaling in commissural neurons (Qu et al., 2013b). Meanwhile, these kinases are also demonstrated to be involved in netrin signaling in primary cortical neurons (Purohit et al., 2012; Qu et al., 2013b). Besides previously mentioned protein kinases, AMPK has also been identified in netrin-Dscam signaling pathway (Zhu et al., 2013). Although the involvement of multiple protein kinases has been identified, the signal transduction mechanisms of Dscam in vertebrates are still poorly understood. Furthermore, research has also reported that in the presence of DCC, netrin-1 is capable of promoting the local translation of *Dscam* mRNA within the growth cones of mouse hippocampal neurons, thereby regulating axon growth and guidance (Jain and Welshhans, 2016).

Furthermore, Dscam can promote axon growth in a netrin-independent manner. This is supported by findings that simultaneous genetic knockout of *Dscam* and netrin receptor fra produces a stronger midline crossing defect than the removal of netrin receptor (Andrews et al., 2008). However, the details for Dscam’s role in netrin-independent signaling remain to be explored. Studies showed that Dscam1 can also mediate axon guiding by acting as a receptor of Slit, a major midline repellent for axons (Alavi et al., 2016; Dascenco et al., 2015).

#### 2.2.3 Dscam isoform diversity

In *Drosophila*, alternative splicing of *Dscam1* gene potentially generates 38,016 isoforms. Previous studies tested the function of single Dscam isoforms in MS, da and MB neurons, and found that they are sufficient to promote self-avoidance at the individual cell level (Matthews et al., 2007; Chen et al., 2006). Although expression of random single isoforms can partially rescue the loss-of-function phenotypes in axonal extension of MS neurons, the specific connection defects remain (Chen et al., 2006). Manipulation of the Dscam1 isoform pool in neurons severely disrupts axonal branching (Hattori et al., 2007; He et al., 2014). These findings suggest that *Drosophila* Dscam isoform diversity is crucial for the function in neural wiring, which cannot be explained by a self-avoidance mechanism. Further studies suggest that Dscam mediates neural wiring during development by tuning the isoform diversity, composition and expression levels.

Multiple studies examined the precise extent to which Dscam isoform diversity is required for neuronal branch phenotypes in three types of neurons. It has been shown that reducing the potential diversity of Dscam to 22,176 isoforms can result in an increase in the variability of axon branching and the appearance of some ectopic branches in a MS neuron (Chen et al., 2006). In the MB neurons, repertoires of 396 and 576 Dscam1 isoforms failed to support the normal patterning of axonal branches, while mutants encoding 1,584 isoforms manifested MB phenotype defects only sporadically (Dong et al., 2022). In another study, scientists generated mutant animals encoding 12, 24, 576, 1,152, 4,752, and 14,256 potential isoforms, and then assessed their effect on branching patterns in da, MB, and posterior scutellar neurons. The results revealed that branching phenotypes are improved as the potential number of isoforms is increased, and at least 4,752 potential isoforms are required for ensuring indistinguishable phenotypes from wild-type controls (Hattori et al., 2009). These data are further examined by using a more comprehensive repertoire of fly mutants harboring reduced isoform diversity, in which the potential ectodomain isoforms gradually decreased from a maximum number of 18,612 to a minimal number of 396 (Dong et al., 2023). This study suggested that, rather than thousands of isoforms, up to 10,000 isoforms may be required for normal axon patterning in MB and MS neurons through a phenotype-diversity correlation analysis (Dong et al., 2023). Notably, this discrepancy is potentially attributed to the small number of deletion mutants analyzed and the lack of mutants generated by partial deletion of exon 6 or exon 9 clusters in the earlier study (Dong et al., 2023). In contrast to MB and MS neurons, da neurons only require approximately 2,000 isoforms for normal dendrite patterning (Dong et al., 2023). These findings indicate that different types of neurons may have a different requirement of isoform numbers for normal patterning.

Previous studies also presented evidence to support the potential phenotypic effect of isoform composition in multiple types of neurons. It is reported that *Dscam* alleles with a moderate reduction in isoform diversity but differing in isoform composition, can cause a different spectrum of abnormalities in the complex branching patterns of a mechanosensory neuron (Chen et al., 2006). To rule out the impact of reduced isoform numbers, scientists further generated a series of allelic cis mutations in *Dscam1*, which encode a normal number of isoforms but with altered isoform composition. Subsequently, they do observe strikingly distinct spectra of phenotypic defects in MB, da, and MS neurons (Zhang et al., 2023). Furthermore, both MB and MS neurons are susceptible to specific exon clusters or isoforms for axon patterning. For example, variations in defective MB phenotypes are more responsive to reductions in the exon 9 cluster than in exon 4 and 6 clusters (Dong et al., 2022; Dong et al., 2023). However, da and posterior scutellar neurons are independent of exon clusters or isoforms (Dong et al., 2022; Dong et al., 2023). Collectively, these findings implicate that diverse neurons may differ in the splicing-tuned regulation strategy for neurite growth, a mechanism of biased usage of variable exons during development (Sun et al., 2013).

Experiments regarding the functional interaction between expression level and isoform diversity of Dscam further deepen the understanding of phenotypic effect mediated by Dscam isoform diversity. When the isoform number of Dscam is reduced, the various phenotypic defects can be improved with decreasing levels of Dscam expression in da, MB, and MS neurons (Dong et al., 2022; Dong et al., 2023). In contrast, while the isoform composition is changed, reducing Dscam1 dosage could exacerbate axonal defects in MB and MS neurons, but revert the dendritic branching and growth defects in da neurons (Zhang et al., 2023). These results reveal a shared and distinct effect of lowering Dscam1 levels on phenotypes between isoform diversity and composition. Therefore, it is speculated that Dscam1 isoform composition may function through a mechanism distinct from isoform diversity. Available evidence suggests that changes in isoform abundance can impair the ability of axon branching in response to dosage of Dscam (He et al., 2014).

Besides diverse ectodomain isoforms, *Drosophila* Dscam contains two alternative TM segments, derived from exon 17.1 and exon 17.2, respectively. It is reported that *Dscam* alleles containing one of the two exon 17 are crucial for the subcellular localization of Dscam, and subsequently affect morphogenesis of axons and dendrites (Shi et al., 2007; Wang et al., 2004; Zhan et al., 2004; Liu H. et al., 2020). For example, *Dscam* transgene that possesses exon 17.2 preferentially targets to axons and its deletion selectively blocked axon arborization, which cannot be rescued by Dscam containing exon 17.1 (Shi et al., 2007; Wang et al., 2004; Zhan et al., 2004).

The endodomain of *Drosophila* Dscam also exhibits diversity during neural development, governing different stage-specific neuronal morphogenesis. It has been shown that Dscam with full-length endodomain is largely restricted to embryogenesis, while Dscam lacking exon 19 and 23 is mostly presented at postembryonic stages. It is revealed that Dscam lacking exon 19 can effectively targets to neurites, therefore playing an important role in axon bifurcation in neurons (Yu et al., 2009).

### 2.3 Synaptic specificity and plasticity

Dscam has been implicated to play a crucial role in synaptogenesis during development. Analysis of mice carrying a spontaneous mutation revealed that branching of layer V pyramidal neuron dendrites is impaired during the early postnatal stage, correlated with increased apical branch spine density and altered spine morphology (Maynard and Stein, 2012). As neural development proceeds, the defects in dendrite branching and spine density will spontaneously reverse while changes in spine morphology persist (Maynard and Stein, 2012). Several studies demonstrated that Dscam deficiency in pyramidal neurons led to increased dendritic spine maturation (Chen et al., 2022; Maynard and Stein, 2012). To understand the role of Dscam in synaptogenesis, scientists assayed the developmental localization of Dscam in mouse retina. During early stages of retinal development, Dscam is diffusely distributed throughout mouse retinal neurites. As development progresses, it adopts a punctate distribution pattern colocalized with junction markers catenin, which persists into adulthood (de Andrade et al., 2014a). Further study revealed that the Dscam punctate does not colocalize with any synaptic markers, with the exception of synaptogenesis periods (de Andrade et al., 2014a). During synaptogenesis, Dscam displayed a strong colocalization with several synaptic markers, such as PSD95, SAP102 (de Andrade et al., 2014a).

The role of Dscam in synaptogenesis is further demonstrated by using an *Aplysia* neuronal culture system, in which a sensory neuron only makes synapses with a selective postsynaptic motor neuron. It is found that both pre-and post-synaptic Dscam are dispensable for the emergence of synaptic transmission and clustering of AMPA-like receptors, implicating that the transsynaptic interaction of Dscam is capable of promoting synaptic specificity (Li et al., 2009). In *Drosophila*, Dscam2 generates two isoforms through a cell-type-specific alternative splicing. In the nociceptive circuit, these two isoforms are expressed either presynaptically or postsynaptically to precisely match their synaptic partners in a complementary manner (Galindo et al., 2023). In mice, Dscam controls synapse formation in Purkinje cells by its intercellular association with astrocyte proteins (Dewa et al., 2024). Taken together, these findings indicated that Dscam promotes synaptic specificity by affecting cell–cell interactions.

The homophilic interaction seems a main way for Dscam to affect cell–cell interactions. For example, the homophilic interaction between axon Dscam and extracellular Dscam is reported to guide axon growth. A study showed that Dscam mutation impairs the axon growth of retinal ganglion cells toward their targets (Bruce et al., 2017). In the *Drosophila* olfactory system, Dscam mediates homophilic repulsion for inter-axonal recognition, thereby preventing premature recognition among sensory axons of ORNs (Goyal et al., 2019). As a result, axons of Dscam mutant ORNs terminate at ectopic sites as they project into the antennal lobe (Hummel et al., 2003; Goyal et al., 2019). Furthermore, Dscam is reported to mediate homophilic adhesion for retina cells to stratify their process appropriately in the inner plexiform layer (IPL) of chick retina (Yamagata and Sanes, 2008). Likewise, Dscam determined specificity of lamina targeting for retina cells in the outer plexiform layer of mouse retina (de Andrade et al., 2014b). Collectively, these findings indicate that Dscam mediates targeting specificity through homophilic interaction, thereby promoting synaptic connection during synaptogenesis. Strikingly, the role of Dscam for synaptic specificity relies on homophilic interaction between neurons rather than serving as cell-type-specific recognition cues (Yamagata and Sanes, 2008). During the process of axonal targeting, the code for neuronal recognition can be provided by Dscam diversity in *Drosophila* (Kim et al., 2013).

In addition, Dscam is also implicated in mediating synaptic plasticity for synapse formation during learning process. Both AMPA and NMDA receptors have been identified as crucial in molecular mechanism underlying the process of synaptic plasticity. Study with *Aplysia* neuronal culture system demonstrated that five repeated pulses of 5-HT can induce remodeling of both postsynaptic NMDA and AMPA receptors through Dscam-mediated transsynaptic interaction, correlated with increased colocalization of presynaptic markers (Li et al., 2009). In line with this, our previous study reported that ablation of DSCAM in neurons derived from human induced pluripotent stem cells (iPSCs) could reduce both expression and function of postsynaptic NMDA receptors (Lim et al., 2021). Meanwhile, we do observe a reduced NMDA current in neurons from the anterior cingulate cortex of *Dscam* mutant mice (Lim et al., 2021).

Previous studies also discovered an effect of Dscam on presynaptic terminals to regulate synaptic plasticity. In *Drosophila*, the expression levels of Dscam are instructive for presynaptic terminal growth, which requires Abl activity (Kim et al., 2013; Sterne et al., 2015). In addition, *Drosophila* Dscam2 is reported to suppress synaptic strength through a PI3K-dependent endosomal pathway (Odierna et al., 2020). Consistently, excessive Dscam levels in the mouse model of DS are correlated with GABAergic innervation of neocortical pyramidal neurons (Liu et al., 2023).

**Figure 1:**
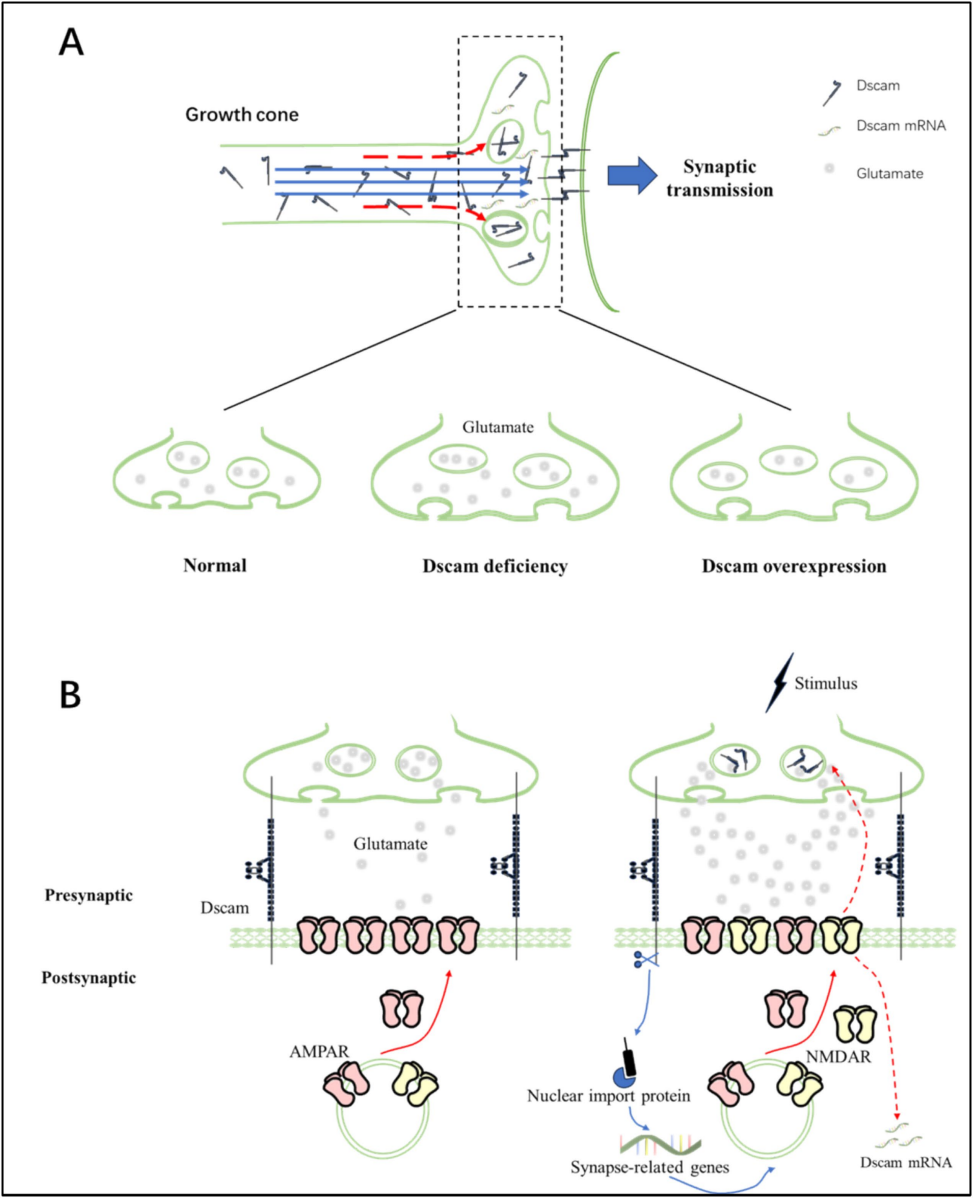
The mechanisms of Dscam for controlling synaptic plasticity. **(A)** Schematic diagram elucidating the local mechanism by which Dscam mediates homeostatic synaptic plasticity at presynaptic sites. At the growth cones of axons, the preponderant amount of Dscam migrates and becomes anchored to the postsynaptic elements, thereby facilitating synaptic transmission (depicted by blue arrows) (Li et al., 2009). Meanwhile, a residual small fraction of Dscam is retained locally within the presynaptic varicosities, serving to modulate the stability of these presynaptic structures (indicated by red arrows) (Li et al., 2009). Notably, both Dscam deficiency and overexpression scenarios are correlated with enlarged presynaptic terminals, albeit with differential impacts on glutamatergic transmission. In the case of Dscam deficiency, due to the compromised regulation of presynaptic varicosity stability, there is an upsurge in spontaneous glutamatergic transmission and accelerated neuronal maturation (Chen et al., 2022; Kim et al., 2013). Conversely, Dscam overexpression leads to an impairment of effective glutamatergic transmission (Liu et al., 2023). Additionally, the local translation of presynaptic Dscam constitutes an essential mechanism underpinning axon outgrowth (Montesinos, 2017). **(B)** Schematic diagram explicating the local mechanism through which Dscam mediates homeostatic synaptic plasticity at postsynaptic sites. Dscam is positioned on the membrane surfaces of both pre-and post-synaptic compartments. It establishes trans-synaptic connections by promoting the clustering of AMPA receptors (AMPAR) at the postsynaptic membrane (as illustrated on the left). Upon stimulation, the membrane localizations of both AMPAR and NMDA receptors (NMDAR) increase within the postsynaptic compartment, a process regulated by Dscam (depicted on the right) (Li et al., 2009). Subsequent to the trans-synaptic interaction of Dscam, its intracellular domain is cleaved and released into the cytoplasm. Subsequently, it is efficiently transported into the nucleus by specific nuclear import proteins, ultimately resulting in altered expression of synapse-related genes (represented by blue arrows) (Sachse et al., 2019). Furthermore, the alterations in postsynaptic NMDARs driven by Dscam would initiate the remodeling of presynaptic Dscam as well as the local translation of postsynaptic Dscam, thereby regulating synaptic plasticity to maintain homeostasis (indicated by red dashed arrows) (Li et al., 2009).

Local regulatory mechanisms may play a crucial role for Dscam to regulate synaptic plasticity. It is proposed that presynaptic and postsynaptic Dscam may mediate different synaptic changes through local regulatory mechanisms (Figure 1). For example, axon-enriched Dscam causes a dramatic increase in presynaptic arbor growth rather than changes in dendrite growth (Kim et al., 2013). Likewise, the *Aplysia* study argued that the introduction of long-term facilitation (LTF) requires postsynaptic Dscam for remodeling of AMPA receptors during learning-related synapse formation, while triggering presynaptic remodeling of Dscam for formation of varicosities and sprouting of axonal filopodia (Li et al., 2009). With the *Aplysia* study as a representative, researchers describe some of local mechanisms. The presynaptic *Dscam* mRNA is concentrated in axonal growth cones, and can be locally translated into functional forms (Jain and Welshhans, 2016; Li et al., 2009). The majority of presynaptic Dscam typically migrates to the postsynaptic processes to anchor growth cones, while a small remainder is retained locally in the presynaptic varicosities (Li et al., 2009). During *de novo* synapse formation, these small portion of presynaptic Dscam may confer neuronal specificity at selected presynaptic varicosities, thereby stabilized at specific postsynaptic sites (Li et al., 2009). During learning related synapse formation, the presynaptic *Dscam* mRNA resided in terminal varicosities is redistributed through dissolution and accumulation. The enrichment of Dscam in the newly formed varicosities appears to stabilize these presynaptic structures at defined sites, thereby allowing the outgrowth of new filopodia and formation of new synaptic connections (Li et al., 2009). On the other hand, the postsynaptic *Dscam* mRNA also induces plastic changes in response to appropriate stimulus. Prior to stimulation, postsynaptic Dscam in innervated neurons could promote the recruitment of glutamate receptors, especially AMPA receptors (Li et al., 2009). In the study of mouse hippocampal neurons, researchers found that the dendritic Dscam is locally translated in response to NMDA application, leading to dendritogenesis (Alves-Sampaio et al., 2010).

Although the knowledge of Dscam-mediated local regulatory mechanisms remain preliminary, available results lead to the speculation that the local mechanisms make up homeostatic synaptic mechanisms, and keep neurons in a functional range of plasticity (Colameo and Schratt, 2022). During early postnatal development, Dscam is firstly colocalized with junction markers catenin, suggesting that it initially functions at intercellular surface to promote spatial organization. As neural development proceeds, the expression levels of Dscam are dramatically increased, accompanied by a progressively significant role in neural wiring. The timing of Dscam’s colocalization with multiple synaptic proteins coincides with a period of synaptogenesis. The presence of Dscam in the active zone of synapse may provide possibility for cellular interaction. During *de novo* synapse formation, the Dscam-mediated cellular interactions induce postsynaptic remodeling of AMPA receptors, resembling a core mechanism of homeostatic synaptic plasticity (Fox and Stryker, 2017). As a result, neurons are capable to be sensitive in response to stimulation, facilitating optimal information processing. The local synaptic adaptations would be conductive to generate network-wide changes, thereby stabilizing the activity of neural networks. Despite a large amount of information about Dscam’s neurological functions, the knowledge of its downstream signaling is little. Available evidence suggested that Dscam may impact synaptic plasticity by translocating its intracellular domain into the nucleus (Sachse et al., 2019).

As the homeostatic plasticity works in an opposite direction to the stimulation, the property of restriction in Dscam’s function gains special attention. In *Drosophila*, elevating Dscam expression in pSC MS neurons could produce specific axonal targeting errors, while MB neurons with Dscam deficiency display additional branches without stereotyped targets (Wang et al., 2002; Cvetkovska et al., 2013). Cortical neurons from DS mice failed to extend their neurite processes and branching, while conditional deletion of Dscam in mouse OFF-type retinal bipolar cells allows dendrite and axon arbors to extend beyond the boundaries of normal tiled fields (Pérez-Núñez et al., 2016; Simmons et al., 2017). Furthermore, Dscam deficiency in mice causes excessive spine maturation and glutamatergic transmission (Chen et al., 2022). Increased dendritic growth and branching rates caused by Dscam deficiency are also observed in Xenopus visual system (Santos et al., 2018). These data indicate that Dscam likely acts as a repressor for spatial organization and synaptic transmission of the developing and mature neurons, enforcing the view of involvement of Dscam in the process of homeostatic synaptic plasticity.

## 3 Dscam-mediated synaptic plasticity and neurological diseases

**Figure 2:**
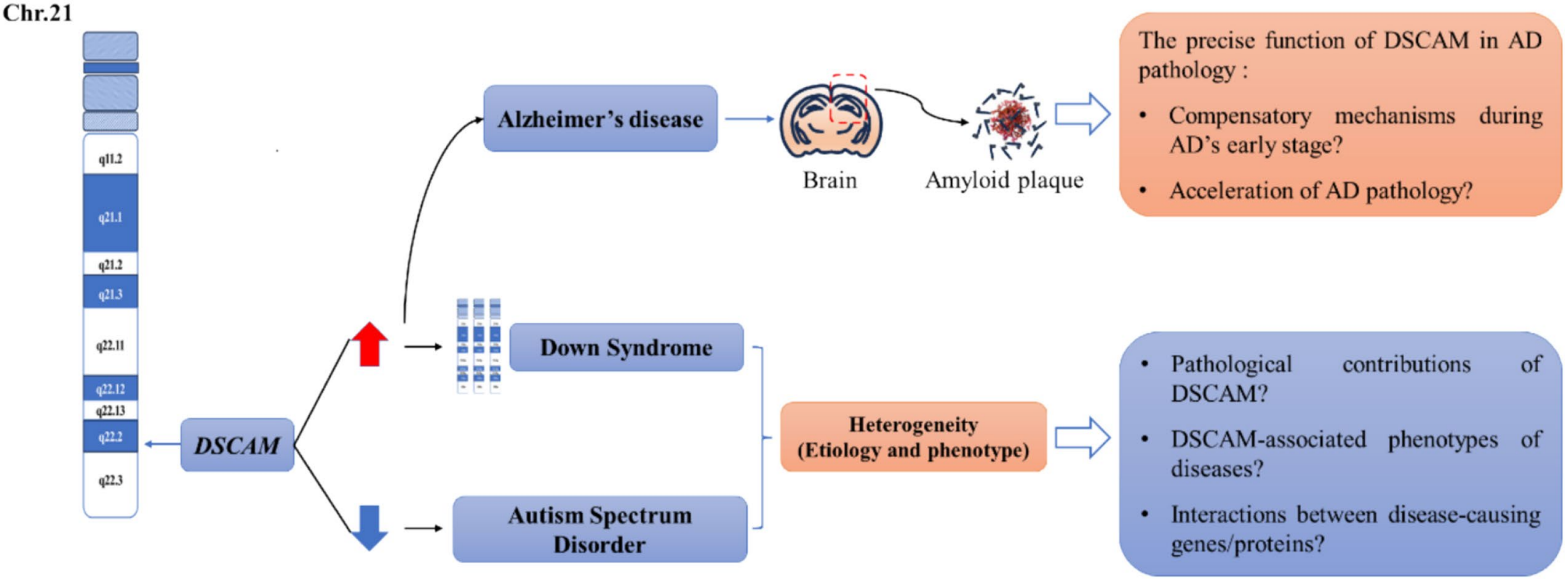
Unresolved questions between Dscam and several common neurological diseases. The human DSCAM gene is located on chromosome band 21q22.2-22.3. Studies showed that aberrant DSCAM expression may be responsible for the cognitive deficits in a subset of patients with Down syndrome (DS) and autism spectrum disorders (ASD). Generally, the expression of DSCAM is elevated in the patients of Down syndrome whereas downregulated in the patients of autism spectrum disorder. Both diseases are heterogeneous in etiology and phenotype, leading to several unresolved issues in this research area, including the pathological contributions of DSCAM? DSCAM-associated phenotypes of disease? Interactions between disease-causing gene/proteins? In addition, Dscam is also reported to be increased in the brain region of patients with Alzheimer’s disease (AD) and corresponding mouse models. Particularly, immunoreactivity for DSCAM is localized to regions associated with senile plaque formation, implicating that DSCAM may involve in the early stage of AD pathology. However, whether DSCAM mediates compensatory mechanisms during AD’s early stage or accelerates AD pathology is still unclear. We argue that a profound understanding of Dscam-mediated homeostatic plasticity can offer crucial insights into how DSCAM contributes to cognitive deficits in these diseases, thereby advancing our ability to tackle these unresolved questions.

Dscam is definitely involved in the process of homeostatic synaptic plasticity. The information about Dscam-mediated homeostatic plasticity is diffusely littered in the current studies. However, there is limited information to elucidate how Dscam induces changes of homeostatic plasticity to affect cognitive function. Dysregulation of Dscam has been implicated in several neurological diseases. The insight into homeostatic plasticity is seriously overlooked in the area of cognitive deficits correlated with aberrant Dscam expression. We argue that this insight would provide an in-depth understanding of the relationship between the neurological basis of cognitive function and pathogenesis of cognitive deficits. Both DS and ASD are representative neurological disorders with aberrant Dscam expression. Furthermore, recent studies have implicated a novel association between Dscam and AD. All these neurological diseases pose significant therapeutic challenges. Therefore, we selected three neurological disorders and focused on homeostatic plasticity to discuss the unresolved questions in this area (Figure 2).

### 3.1 Down syndrome

DS is a chromosomal disorder that results from an extra complete or partial copy of human chromosome 21. The symptoms of DS, which are polygenic and heterogeneous, are primarily determined by the interactions of overexpressed genes (Grossman et al., 2011; Pizzo et al., 2021; Hergenreder et al., 2024). The gene *DSCAM* was initially discovered when identifying genes located in the DS critical region. Significantly, the protein levels of DSCAM are elevated in DS patients (Stachowicz, 2018). It has been demonstrated that the overexpressed DSCAM is likely related to intellectual disability, which is the most prominent feature of DS. The underlying mechanisms are mainly associated with pathways such as neurogenesis and synaptic plasticity (Huo et al., 2018; Liu et al., 2023; Kleschevnikov et al., 2012; Li et al., 2015; Pérez-Núñez et al., 2016; Tang et al., 2021). Nevertheless, despite a handful of studies, the full extent to which Dscam-mediated homeostatic synaptic plasticity is impaired remains largely uncharted territory. For illustration, in a normal situation, the dendritic *Dscam* can be locally translated in response to NMDA application, and this process plays a vital role in regulating synaptic transmission. Nevertheless, this function is compromised in DS (Alves-Sampaio et al., 2010). Furthermore, in DS mice, the overexpressed Dscam could lead to the overgrowth of presynaptic terminals of interneurons and excessive GABAergic transmission (Liu et al., 2023; Kleschevnikov et al., 2012). Studying genetic interactions is more conductive to shedding light on the association between genetic etiology and phenotypes of DS. By means of large-scale Y2H experiments and PLA approaches, researchers have identified an interaction between *DSCAM* and *DYRK1A* in the dendrite, expecting to regulate a synaptic network (Viard et al., 2022). These research findings are instrumental in deepening our comprehension of Dscam’s role in DS etiology and phenotypes. Hence, it is of paramount importance to conduct further in-depth investigations into Dscam-mediated homeostatic synaptic plasticity to unlock potential therapeutic avenues and a more profound understanding of the disorder.

### 3.2 Autism spectrum disorder

ASD, a heritable yet etiologically intricate neurodevelopmental disorder, is predominantly typified by impaired social communication, repetitive and inflexible behaviors. It is commonly recognized that variations in ASD-risk genes and mutations govern the susceptibility, heterogeneity of autistic symptoms among ASD patients, as well as their diverse responses to interventions (De Rubeis et al., 2014; Wang et al., 2016; Turner et al., 2016; Iossifov et al., 2014; Yuen et al., 2017; Narita et al., 2020; Satterstrom et al., 2020; Yuan et al., 2023). Notably, *DSCAM* mutations show a relatively high frequency in ASD patients, mostly as protein-truncating variants (PTVs) (Wang et al., 2016; Turner et al., 2016; Yuen et al., 2017; Satterstrom et al., 2020). The link between *de novo DSCAM* loss-of-function mutations and ASD is further buttressed by the observation of autistic-like behaviors in Dscam-deficient mice (Chen et al., 2022; Lim et al., 2021; Neff et al., 2024). Furthermore, it has been discovered that ASD individuals harboring *DSCAM* PTVs chiefly present with developmental delay and intellectual disability (Lim et al., 2021; Wang et al., 2016; Narita et al., 2020). Despite the underlying mechanisms remain shrouded in mystery, current study has linked Dscam to the process of neuronal communication in ASD pathology (Satterstrom et al., 2020). Reports show Dscam is abundant in cortical excitatory neurons, and its deficiency boosts glutamatergic transmission in the developing cortex, leading to ASD-like behaviors in mice (Chen et al., 2022; Satterstrom et al., 2020). However, our previous study found Dscam ablation reduced NMDA receptor and post-synaptic NMDA currents in ASD iPSC-induced glutamatergic neurons (Lim et al., 2021). This seeming contradiction could potentially be ascribed to Dscam-mediated homeostatic synaptic plasticity. In cases of Dscam deficiency, the resultant neuronal hyperactivity elevates the firing threshold, rendering neurons unresponsive to further stimulation. As *DYRK1A* is a high-risk gene for ASD, previously identified genetic interaction between *DSCAM* and *DYRK1A* in DS mice sheds light on a link between DS and ASD, implicating an association of DSCAM with cognitive deficits in ASD (Viard et al., 2022). Consequently, investigations into Dscam-mediated homeostatic synaptic plasticity will be conducive to integrating existing findings and future discoveries regarding the underlying mechanisms of ASD pathology, thereby deepening our understanding of the etiological and phenotypic diversity of ASD.

### 3.3 Alzheimer’s disease

AD is a neurodegenerative disease marked by progressive cognitive and behavioral problems. Remarkably, individuals with DS are more susceptible to developing early-onset AD later in life, although the principal cause of cognitive decline in AD is the overexpression of *amyloid precursor protein (APP)* (Hergenreder et al., 2024). It is widely acknowledged that studying AD via the DS model would help track its onset and decipher preclinical mechanisms (Martinsson et al., 2022; Head et al., 2007). Research indicates in DS patients with AD, DSCAM expression is localized to regions associated with senile plaque formation (Head et al., 2007). Moreover, in APP transgenic mice, the level of Dscam expression in the cerebral cortex and hippocampus was significantly elevated and progressively increased with age (Jia et al., 2017; Jia et al., 2011). Recent comprehensive big data analyses have further spotlighted *Dscam* as a candidate gene contributing to the genetic predisposition for early-onset AD (Li et al., 2023; Niu et al., 2023). Therefore, it’s plausible that Dscam might be one of the key molecules involved in neuronal age-related compensatory mechanisms during AD’s early stage. However, regrettably, only limited research has focused on clarifying the precise function of Dscam in AD pathology, an area that urgently demands the attention of the scientific community. There appears to be an intricate functional interplay between APP and Dscam, as the *Drosophila* homolog of APP promotes Dscam expression post-transcriptionally to drive axon growth (Pizzano et al., 2023). Given disrupted synaptic homeostasis with amyloid-β (Aβ), several scientific questions arise: whether Aβ-induced neurotoxicity is the direct cause of elevated expression of Dscam? Whether Dscam accelerate AD pathology by decreasing resistance to Aβ-dependent neuronal hyperactivity? How Dscam-mediated homeostatic synaptic plasticity works in AD pathology? With future rigorous investigations, these crucial questions will be meticulously and progressively dissected, holding great promise for the development of novel treatments for AD.

## 4 Conclusion

In the current review, we present comprehensive evidences highlighting the crucial role of homeostatic regulation on neural network mediated by Dscam. For illustration, it functions pivotally in modulating neuronal migration, neuronal morphology and branch patterning, as well as synaptic specificity and plasticity. This is generally achieved via homophilic interactions, adhesive mechanisms and isoform diversity. Through these functions, Dscam endows the neural network with a remarkable homeostatic regulatory capacity, and then empowers neurons to proficiently process information, even when confronted with environmental disruptions to neural activity. By employing DS, ASD, and AD as illustrative cases, we contend that Dscam plays an essential role in cognitive processes through regulating homeostatic neural plasticity. Future investigations focused on unraveling the profound significance of Dscam in relation to cognitive deficits will undoubtedly pave the way for precisely targeting Dscam as a tangible strategy to combat such deficits.
